# Supplementary figures and images for: Genome-Wide Identification and Functional Characterization of FAR1-RELATED SEQUENCE (FRS) Family Members in Potato (Solanum tuberosum)
Source: Plants (Basel). 2023 Jul 7;12(13):2575. doi: 10.3390/plants12132575 (PMC10347153; doi:10.3390/plants12132575)

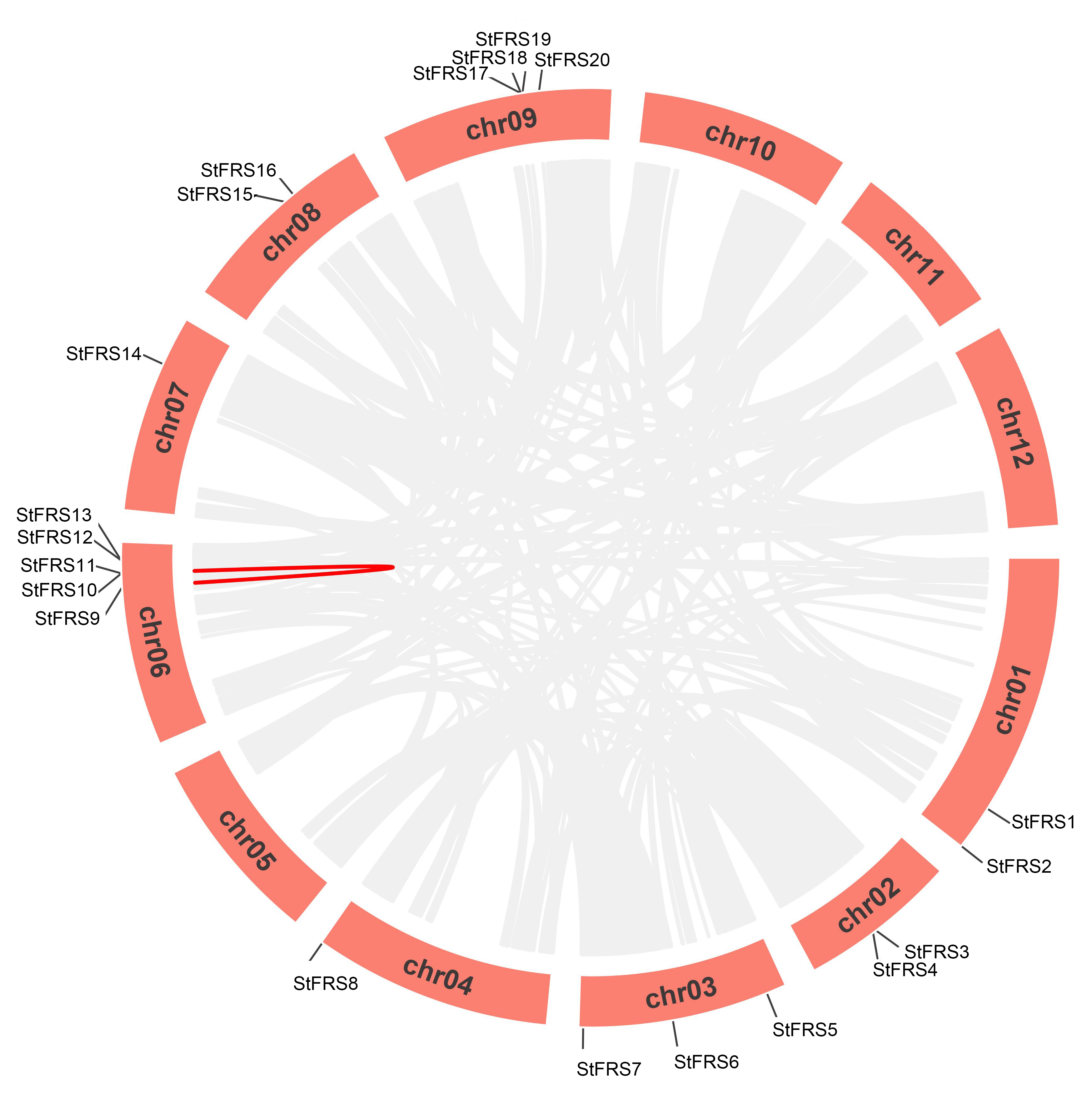

Supplement: Supplementary file 1 [file plants-12-02575-s001.zip › Figure S1. Synteny analysis of StFRSs in potato genome.jpg]

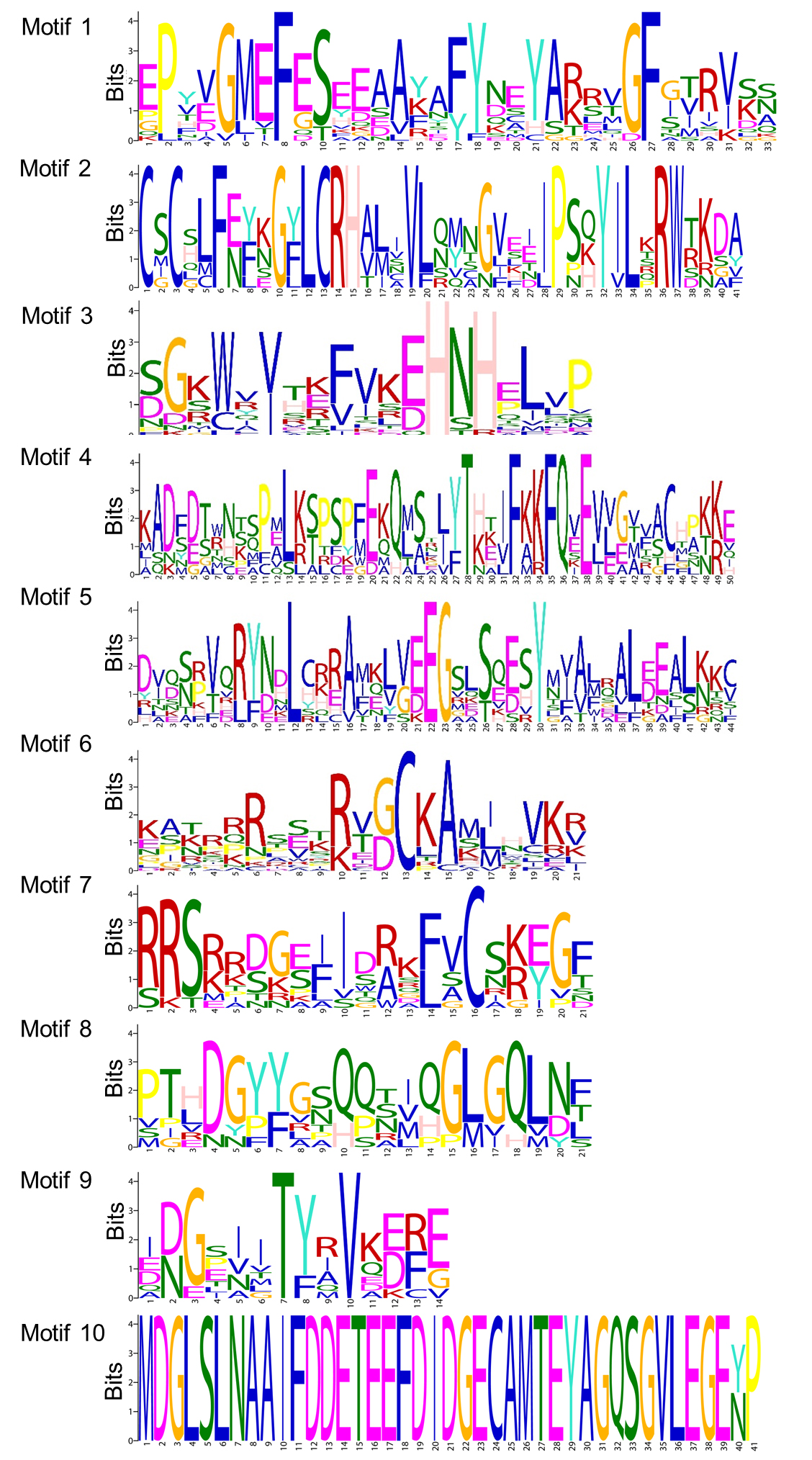

Supplement: Supplementary file 1 [file plants-12-02575-s001.zip › Figure S2. Motif sequences identified for StFRS proteins.jpg]
